# A Machine Learning Approach for Spatial Mapping of the Health Risk Associated with Arsenic-Contaminated Groundwater in Taiwan’s Lanyang Plain

**DOI:** 10.3390/ijerph182111385

**Published:** 2021-10-29

**Authors:** Ching-Ping Liang, Chi-Chien Sun, Heejun Suk, Sheng-Wei Wang, Jui-Sheng Chen

**Affiliations:** 1 Department of Nursing, Fooyin University, Kaohsiung City 831, Taiwan; sc048@fy.edu.tw; 2 Graduate Institute of Applied Geology, National Central University, Taoyuan City 320, Taiwan; vincent10308211@gmail.com; 3 Korea Institute of Geoscience and Mineral Resources, Daejeon 34132, Korea; sxh60@kigam.re.kr; 4 Department of Water Resources and Environmental Engineering, Tamkang University, New Taipei City 251, Taiwan; wangsw@mail.tku.edu.tw

**Keywords:** back-propagation neural network, ordinary kriging, groundwater arsenic contamination, hazard quotient, target risk

## Abstract

Groundwater resources are abundant and widely used in Taiwan’s Lanyang Plain. However, in some places the groundwater arsenic (As) concentrations far exceed the World Health Organization’s standards for drinking water quality. Measurements of the As concentrations in groundwater show considerable spatial variability, which means that the associated risk to human health would also vary from region to region. This study aims to adapt a back-propagation neural network (BPNN) method to carry out more reliable spatial mapping of the As concentrations in the groundwater for comparison with the geostatistical ordinary kriging (OK) method results. Cross validation is performed to evaluate the prediction performance by dividing the As monitoring data into three sets. The cross-validation results show that the average determination coefficients (R^2^) for the As concentrations obtained with BPNN and OK are 0.55 and 0.49, whereas the average root mean square errors (RMSE) are 0.49 and 0.54, respectively. Given the better prediction performance of the BPNN, it is recommended as a more reliable tool for the spatial mapping of the groundwater As concentration. Subsequently, the As concentrations estimated obtained using the BPNN are applied to develop a spatial map illustrating the risk to human health associated with the ingestion of As-containing groundwater based on the noncarcinogenic hazard quotient (HQ) and carcinogenic target risk (TR) standards established by the U.S. Environmental Protection Agency. Such maps can be used to demarcate the areas where residents are at higher risk due to the ingestion of As-containing groundwater, and prioritize the areas where more intensive monitoring of groundwater quality is required. The spatial mapping of As concentrations from the BPNN was also used to demarcate the regions where the groundwater is suitable for farmland and fishponds based on the water quality standards for As for irrigation and aquaculture.

## 1. Introduction

Groundwater accounts for a substantial portion of the freshwater supply in the Lanyang Plain, Taiwan. To resolve the problem of a lack of reservoirs for the storage of seasonal rainfall and the poor quality of the surface water, area residents are heavily reliant upon the groundwater for agricultural irrigation, aquaculture, domestic and drinking purposes. Groundwater quality monitoring for the Lanyang Plain conducted by the Environmental Protection Bureau (EPB) of Yilan County [1,2,3] has clearly identified that the arsenic (As) content in some monitoring wells exceeds the World Health Organization’s (WHO) permissible drinking water threshold of 10 µg/L [4]. Arsenic has been classified as a Group 1 carcinogen by the International Agency for Research on Cancer (IARC) [5]. The primary exposure pathway of groundwater As is through the ingestion of groundwater. The ingestion of groundwater high in As has adverse effects on human health, leading to many diseases such as cancers, skin lesions, peripheral microvascular disease and Blackfoot disease [6,7,8,9,10]. However, geographical visualization of groundwater As concentrations in the Lanyang Plain shows considerable spatial variability, which means that the associated risk to human health would also be an issue of geographical dependence. Clearly, there is an urgent need to accurately map the substantial geographical variability in groundwater As concentration.

Conventional spatial mapping methods, such as kriging, which is based on geostatistical theory, have been widely used for modeling the spatial variability of groundwater quality variables with limited field data. Lee et al. [11] and Liang et al. [12,13], respectively, applied indicator kriging (IK) and ordinary kriging (OK) techniques to assess the spatial distribution of the carcinogenic and non-carcinogenic health risks related to drinking As-containing groundwater. Jang et al. [14] applied the multivariate indicator kriging (MVIK) to spatially characterize the regions where the groundwater quality is safe for multipurpose utilization in the Pingtung Plain. Liang et al. [15] applied the OK technique for spatial characterization of the regions where groundwater quality is safe for multipurpose utilization in the Pingtung Plain and Lanyang Plain. Despite the geostatistical kriging approach being widely applied to spatially assess the groundwater quality variable, the results of spatial health risks associated with As produced with the kriging technique may not be sufficiently accurate because of the heterogeneity of the hydraulic properties of the aquifer and the nonlinearity of the contaminant transport processes [16].

In contrast, data-driven machine learning techniques, such as artificial neural network (ANN) or random forest (RF) methods, can facilitate the process by resolving a spectrum of nonlinearity problems. Purkait et al. [17] developed a four-layer feed-forward back-propagation neural network (BPNN) model (7-15-15-1), which could be used as an acceptable prediction model for estimating the groundwater As concentrations in Eastern India. Cho et al. [18] applied four different models, namely, multiple linear regression (MLR), principal component regression (PCR), artificial neural network (ANN) and the combination of principal components and an artificial neural network (PC-ANN), for the prediction of potential groundwater As contamination in Southeast Asian countries. The results show that PC-ANN yielded a superior outcome with a significant performance improvement due to the Nash–Sutcliffe model efficiency coefficient (NSE). Chowdhury et al. [16] compared the ANN and ordinary kriging (OK) techniques for spatial estimation of the As concentrations in Bangladesh, and pointed out that a highly nonlinear pattern machine learning technique in the form of an ANN model can yield more accurate results than OK under the same set of constraints. Jeihouni et al. [19] used the OK and two AI methods, namely, ANN and the adaptive neuro-fuzzy inference system (ANFIS), to spatially assess the electrical conductivity of groundwater. Their results indicated that ANFIS provides the best prediction accuracy with a root mean squared error (RMSE) value of 1.69 dS.m, whereas the RMSEs are 1.79 dS.m and 2.14 dS.m for ANN and OK, respectively. Jia et al. [20] performed a comparison study for the estimation of the spatial distribution of regional cadmium and arsenic pollution using the OK and BPNN methods. Their results showed BPNN to have a higher prediction accuracy, with mean square errors (MSEs) of 0.0661 and 0.1743 for As and Cd, respectively, than did OK, with MSEs of 0.0804 and 0.2983 for As and Cd, respectively.

The aforementioned studies illustrate that the machine learning approach has the potential to act as a spatial mapping tool with high prediction performance for several groundwater quality issues. This study is thus designed to develop the ANN as a spatial mapping tool for estimation of the geographical variability of As concentrations in the Lanyang Plain. We also make a comparison between the prediction performance of ANN and the conventional OK method. The predicted As geographical distribution is further used to calculate the noncarcinogenic hazard quotient (HQ) and carcinogenic target risk (TR) and demarcate the regions where people are at a higher health risk. The yielded health risk can be used for improving the decision-making process for health risk management associated with ingestion of As-containing groundwater in the Lanyang Plain.

## 2. Materials and Methods

### 2.1. Study Area

**Figure 1:**
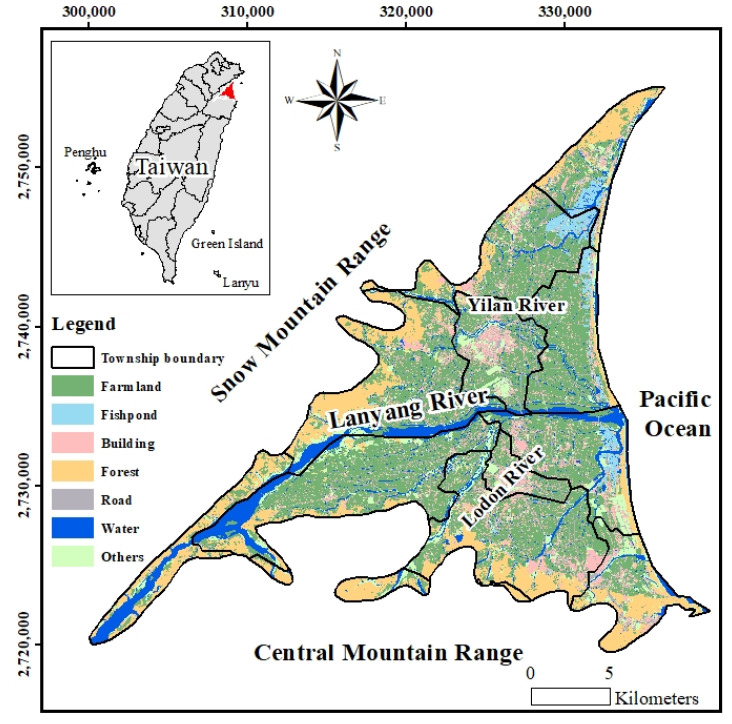
Land use in the Lanyang Plain.

The Lanyang Plain is an alluvial fan on the Lanyang River bound by the Snow Mountains on the northwestern side, the Central Range to the southwest and the Pacific Ocean on the east (Figure 1). The land in the Lanyang Plain is heavily utilized for agriculture with aquaculture along the coast. Because of the lack of large water-storage facilities, the main water supply comes from Luodong and Cukeng Weirs. However, surface water quality is slightly and moderately affected by contamination from household and stock-farming wastewater. Although the coverage of the tap water supply system is up to 90%, most residents still use groundwater from private wells for household purposes. In addition, about 60% of the tap water also originates from groundwater sources.

**Figure 2:**
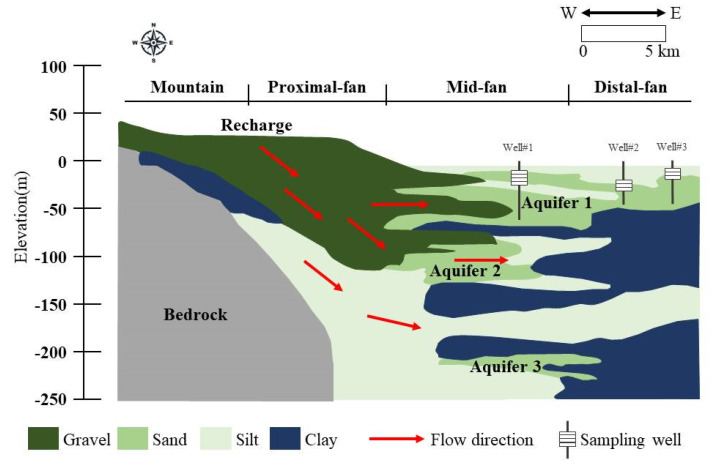
Hydrogeological profile of the Lanyang Plain. Reference Source: [21].

The Lanyang alluvial fan is composed of recent alluvial deposits, including gravel, sand and silt, and clay comprised of detrital slates, quartz sandstone and crystallized gneiss [21]. The bedrock, overlain by the alluvial deposits, is the Suao slate and argillite of the Miocene age, occasionally with a thin layer of metamorphosed sandstone [21]. The subsurface hydrogeology of the Lanyang Plain includes one shallow unconfined aquifer (Aquifer 1) and two underlying confined aquifers (Aquifer 2 and Aquifer 3), as well as two aquitards (Figure 2). The geology material between the proximal area and the center of the fan are coarse sand and gravel; these regions are highly permeable and the primary source of groundwater in the aquifers [22]. The eastern coastal regions which consist mainly of fine sand and the clay is less permeable. Groundwater flow generally follows the surface topography from the western mountains to the eastern coasts. The climate of the area is subtropical with northeasterly monsoon winds blowing when autumn changes to winter. The northeasterly monsoon winds combined with the western mountains produce heavy rainfall in the winter season. In the summer season, convectional rainfall occurs due to the higher temperature. Table 1 summaries the average rainfall and temperature in the Lanyang plain. The abundant rainfall gives the Lanyang Plain a rich supply of groundwater [21].

Most recently, Liu and Wu [22] performed a study on the geochemical, mineralogical and statistical characteristics of arsenic in groundwater of the Lanyang Plain. They concluded that arsenic in sediments is released into groundwater primarily by the reductive dissolution of As-bearing Fe-oxyhydroxides in the reducing environment at Langyang. As concentrations at depths of 100–180 m can achieve the maximum concentration of 900 µg/L [22].

### 2.2. Artificial Neural Network

As a data-driven method, ANNs can learn the complex mapping between the input and the output given sufficient data, and their flexible structure can also provide a good estimation for various problems. ANNs are designed to simulate the process of the transport of electric potentials by neural cells in living creatures. The single neuron operates along the following functions:

(1) $$net_{j}=\sum_{i=1}^{n}X_{i}\cdot W_{ji}-b_{j};$$

(2) $$Y=f\left(net_{j}\right),$$

where $X_{i}$ represents the ith input variable; $W_{ji}$ represents the corresponding weighting factors for the *i*th input variable; $b_{j}$ represents a bias; f() represents an activation function; and *n* is the number of input data.

The structure of an ANN includes three main layers. First, there is an input layer, which is responsible for receiving the input variables and transporting the signal to the next layer without any artificial neurons being used in the computation. Second, there is at least one hidden layer, which is composed of artificial neurons for the computation operation and which is used to extract the patterns associated with the process or system being analyzed. The role of this layer (or these layers) is to perform most of the internal processing in the network. The last output layer is also composed of neurons and is responsible for producing the final network outputs with the same format as the real output value set in the training process [23].

A feed-forward back-propagation neural network (BPNN) was chosen for use in this study. The feedforward BPNN training procedure is a supervised learning method and is divided into two main parts. The Levenberg–Marquardt (LM) algorithm, used for training in this study, is a blend of the gradient descent and Gauss–Newton iterations, and is probably the most widely used optimization method, since its hyper-spherical trust region has proven to provide a better solution in searching for the minima [16].

### 2.3. Ordinary Kriging (OK)

The actual spatial data were mostly messy and scattered, with adjacent data usually having a higher degree of similarity and correlation than those far away. The core of the geostatistical kriging technique is the regionalized variable theory, which states that the variables in an area exhibit both random and spatially structured properties and a second-order stationary process is assumed [24]. A geostatistical variogram was used to characterize the spatial variability between the values of the regional variables at two observation locations. A semi-variogram γ(h) can be mathematically calculated as follows:

(3) $$\gamma \left(h\right)=\frac{1}{2N\left(h\right)}\left\{\sum_{i=1}^{N\left(h\right)}{\left[Z\left(x_{i}+h\right)-Z\left(x_{i}\right)\right]}^{2}\right\},$$

where h denotes the distance between two observation locations; $Z\left(x_{i}\right)$ is the value of the regional variable at the observation location$\;x_{i}$; $Z\left(x_{i}+h\right)$ is the value of the regional variable at the observation location $x_{i}+h$; and N(h) is the number of pairs for two observation locations separated by a distance h.

The experimental semi-variograms were calculated pair-by-pair using Equation (3) and subsequently fitted against a theoretical semi-variogram model of γ(h). The main parameters affected are the range (a), nugget effect ($c_{0}$) and sill ($c+c_{0}$). If there is a considerable change in the concentrations of two observations separated by a small distance, it will produce a nugget effect ($c_{0}$). The widely used theoretical models are written as follows:

Spherical semi-variogram model:

(4) $$\gamma \left(h\right)=\{\begin{array}{c} c_{0}+c\left[1.5\left(\frac{h}{a}\right)-0.5{\left(\frac{h}{a}\right)}^{3}\right]\;\;h\leq a\\ c_{0}+c\;\;h>a \end{array};\;$$

Exponential semi-variogram model:

(5) $$\gamma \left(h\right)=c_{0}+c\left\{1-exp\left[-\left(\frac{3h}{a}\right)\right]\right\};$$

Gaussian semi-variogram model:

(6) $$\gamma \left(h\right)=c_{0}+c\left\{1-exp\left[-{\left(\frac{3h}{a}\right)}^{2}\right]\right\}.$$

Ordinary kriging is a spatial interpolation estimator that is applied to find the best linear unbiased estimate at a non-sampled location$\;x_{0}$ and is determined according to the linear combination of the known values of all the sampled locations as follows:

(7) $$Z^{*}\left(x_{0}\right)=\sum_{i=1}^{M}\lambda_{i}\left(x_{i}\right)Z\left(x_{i}\right),$$

where $Z^{*}\left(x_{0}\right)$ is the unknown value of the regional variable that will be determined at a non-sampled location$\;x_{0}$; $Z\left(x_{i}\right)$ is the known value of the regional variable at a sampled location$\;x_{i}$; *M* is the total number of the sampled locations; and $\lambda_{i}\left(x_{i}\right)$ is a kriging weighting factor for the known value of the random variable$\;Z\left(x_{i}\right)$ at a sampled location $\left(x_{i}\right)$, which is used to determine $Z^{*}\left(x_{0}\right)$.

### 2.4. Health Risk Assessment

This study assesses the health risk, specifically the carcinogenic and non-carcinogenic risks, associated with the drinking of As-contaminated (inorganic) groundwater using the methods recommended by the USEPA [25,26].

The carcinogenic risk is evaluated based on the target risk (*TR*) index, which is used to quantify the cancer risk caused by those substances classified as definite or probable human carcinogens. Thus, an estimated *TR* value equal to $1\times 10^{-6}$ indicate that one additional person out of one million people will suffer from cancer due to these substances in their lifetime. The *TR* (life time risk index) is formulated as follows:

(8) $$TR=\frac{C\cdot IR}{BW}\cdot \frac{EF\cdot ED}{AT}\cdot CSF\cdot 10^{-3},$$

where *C* is the As concentration (µg/L); *IR* is the daily water intake (L/day); *ED* is the exposure duration (year); *EF* is the exposure frequency (day/year), which is how many days an individual is exposed to As over the course of a year; *BW* is the body weight (kg); *AT* is the average life time for carcinogenic exposure (days); *CSF* is the cancer slope factor (mg/L) obtained from the Integrated Risk Information System (IRIS) database; and 10^−3^ is a conversion factor. The cancer slope factor (*CSF*), which is used for characterizing the relationship between dose and response, is a key parameter in the *TR* model. The *CSF* is an upper-bound estimate of the probability that a person will develop cancer when exposed to a chemical over a lifetime of 70 years.

The non-carcinogenic risk is evaluated based on the hazard quotient (*HQ*) index which is defined as the ratio of potential exposure to a reference magnitude for which there are no expected adverse effects. If the *HQ* value is greater than 1, an adverse non-carcinogenic effect is regarded as possible. The *HQ* is calculated by

(9) $$DI=\frac{C\cdot IR}{BW};$$

(10) $$HQ=\frac{DI}{RfD}\;,$$

where *DI* is the daily intake of As (µg/kg/day); *C* is the As concentration (µg/L); *IR* is the daily water intake (L/day); and *RfD* is the oral reference dose derived by the USEPA [26].

## 3. Results and Discussion

### 3.1. Groundwater Monitoring Data and Preprocessing

**Figure 3:**
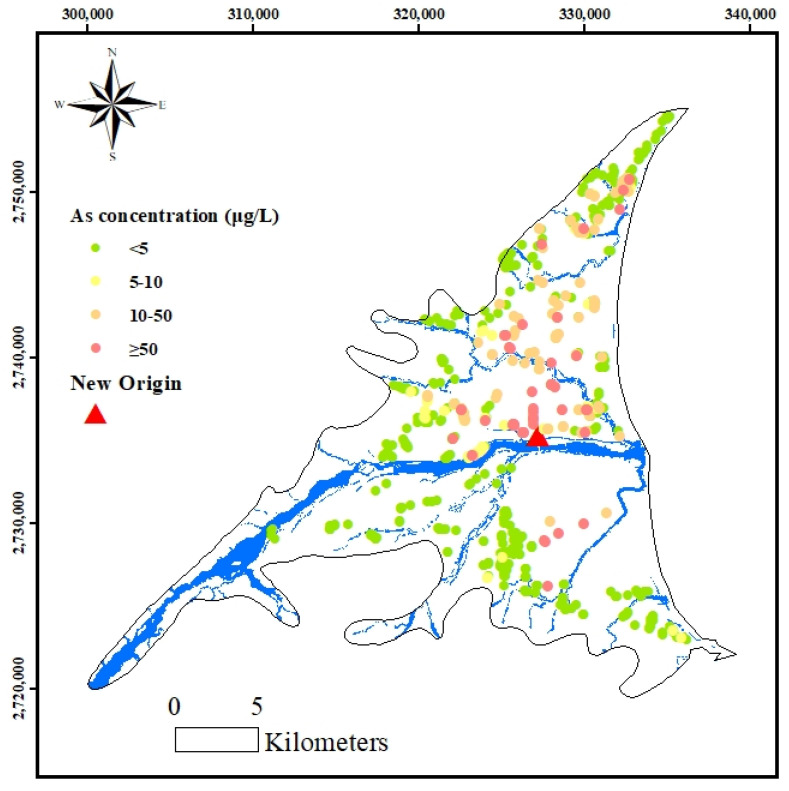
Geographical distribution of the measured As concentrations.

The groundwater monitoring data used in this study were collected from 921 household wells located in Aquifer 1, as shown in Figure 2 (below 40 m in depth), during the period from 1997 to 1999 by the Environmental Protection Bureau (EPB) of the Yilan County Government (EPB, 1997; 1998; and 1999). The survey was carried out as part of a health-related study of the residential wells used to supply drinking water in townships in the Lanyang Plain. Groundwater was pumped out for at least 10 min before sampling in order to obtain a representative sample. Seven water quality items were analyzed, including the As concentration, pH, ammonia, nitrite, nitrate, iron and manganese. Except for pH, which was measured in situ, others were analyzed in the laboratory. The analysis procedures of the As concentrations in the groundwater samples followed the APHA Method 3500-AsB [13]. The area residents have been using shallow wells (<40 m) to obtain drinking water since the 1940s, which means they may have been consuming high-As artesian well water for over 60 years. Figure 3 shows a geographical visualization of the As concentration levels of the 921 samples. The results of a descriptive statistical analysis of the collected As concentration data are summarized in Table 2. The As concentration ranges from below the detection limit (0.9 µg/L) to a maximum value of 772 µg/L. The average concentration is 11.9 µg/L, with a standard deviation of 45.21 µg/L. The water quality standard for the As concentration in the drinking water recommended by the WHO is 10 µg/L in contrast to the 82.75th percentile of the cumulative percentage for the measured As concentrations. The 921 samples were uniformly divided into three sets (labelled A, B and C) in the order of the magnitude of As concentration for the purpose of cross-validation and performance evaluation. Two sets of data were used to construct the BPNN and OK models, while the third was used to validate the constructed BPNN and OK models. In order to group the data sets evenly, the three data sets were distributed based on the concentration levels.

Data processing is an important step in the procedure for optimizing the prediction results obtained with the BPNN and OK methods. To reduce the complexity, the coordinate data were arranged in relation to the approximate center of the total sample locations by setting a new origin (327245, 2735281) (Figure 3). The As concentrations were processed by the application of logarithmic transformation to ensure correspondence to a normal distribution. The *p*-values of the log-transformed concentration and the original concentration are 0.523 and 0, indicating that the logarithmic transformation can efficiently change the data to approximate a normal distribution more closely. For both the BPNN and the OK method, these preprocessing steps are useful for reducing noise in the prediction models.

### 3.2. Arsenic Concentration Prediction

**Figure 4:**
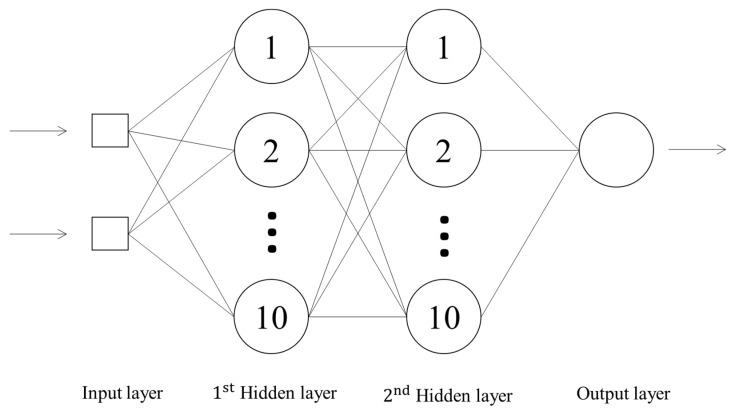
Structure of the BPNN used in this study.

An exponential semi-variogram model (Equation (3)) was applied to fit the experimental semi-variograms data for each individual training dataset of the OK method. Table 3 lists the fitting ranges, nugget effects and sills of each training dataset. A neural network was set up and trained using the back-propagation algorithm. Two nodes in the input layer correspond to the input data (x and y coordinates), and one neuron in the output layer corresponds to the estimated As concentrations. The parameters used in building the BPNN are shown in Table 4. In this study, MATLAB 2019 (MathWorks) was applied to develop the computer code for constructing the BPNN. The convergence criteria used to terminate the training process were set at 10^−2^ of the mean squared error. The structure of the developed model is shown in Figure 4. Determining the number of hidden neurons is usually a matter of trial and error.

Table 5 shows the average values of the coefficients of determination (R^2^) and RMSEs for each different BPNN model tried in this study. Based on the highest average R^2^ value and the lowest average RMSE value, model (2,10,10,1) was chosen to apply for spatial mapping and for comparison of the mapping performance with that obtained from the OK method. Table 6 summarizes the R^2^ and RSME values for BPNN and OK. The results show that the average R^2^ values for cross validation of the As concentrations obtained with BPNN and OK are 0.55 and 0.49, whereas the average RMSE values are 0.49 and 0.54, respectively. Based on the average R^2^ and RSMEs, we can conclude that the BPNN provides better performance than the OK.

### 3.3. Application of Spatial Mapping of the As Concentrations Using BPNN

**Figure 5:**
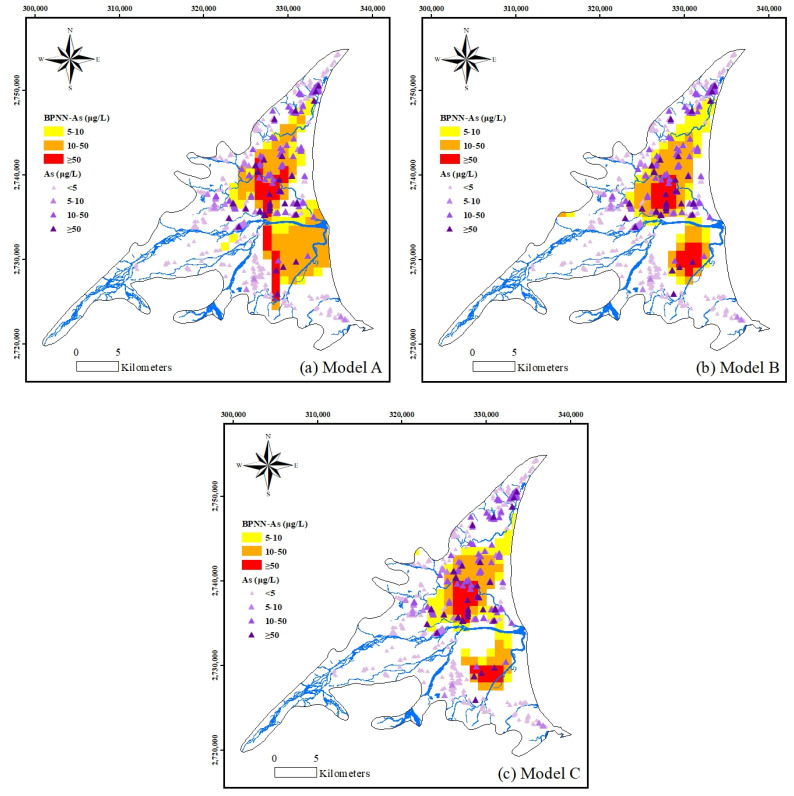
Spatial mapping of unacceptable As concentrations estimated by BPNN.

The BPNN was then used to predict the geographical distribution of As concentrations in the Lanyang Plain. First, the area of the Lanyang Plain was spatially discretized into a grid system of 1 km × 1 km grids. The As concentrations were calculated at each grid center from the output of the BPNN model. Figure 5 shows the geographical visualization of the As concentrations obtained by the BPNN model. The As concentrations were classified into four levels: >5, 5–10, 10–50 and <50 ppb. The measured data are also included in Figure 5, with an identical four-level classification of the concentration.

The As concentrations at each grid center obtained using BPNN were then used to calculate the *TRs* and *HQs* (using Equations (8) and (10)) with which to demarcate the regions of unacceptable carcinogenic and non-carcinogenic risk. The values of the parameters required for assessment of the health risk calculated with Equations (8)–(10) are shown in Table 7.

**Figure 6:**
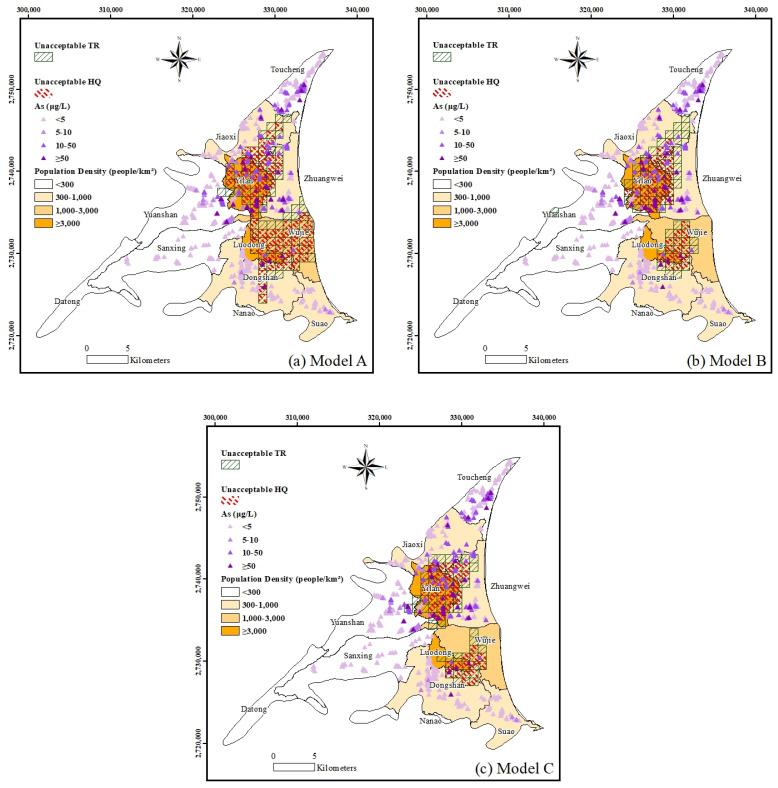
Spatial mapping of unacceptable *HQs* and *TRs* coupled with population density.

The *TRs* are classified into three levels: Level 1, with a *TR* value of less than $1\times 10^{-6}$, which means that there is negligible risk; Level 2, where the *TR* value is between $1\times 10^{-6}$ and $1\times 10^{-4}$, which means that there is an acceptable risk; and Level 3, with a *TR* value of greater than $1\times 10^{-4}$, indicating unacceptable risk. The *HQ* values were classified into two levels: Level 1, in which the *HQ* value is greater than 1, which is considered to cause an adverse non-carcinogenic outcome; and Level 2, where the *HQ* values are lower than 1, which means an acceptable adverse non-carcinogenic outcome. Figure 6 shows the spatial mapping of the unacceptable *TRs* and *HQs*, which could result in carcinogenic and non-carcinogenic risk. Together with the distribution of population density, the map can define the areas of high-risk groundwater usage. According to Figure 6, it is advised that groundwater is not suitable for drinking in the townships of Yilan and Luodong.

**Figure 7:**
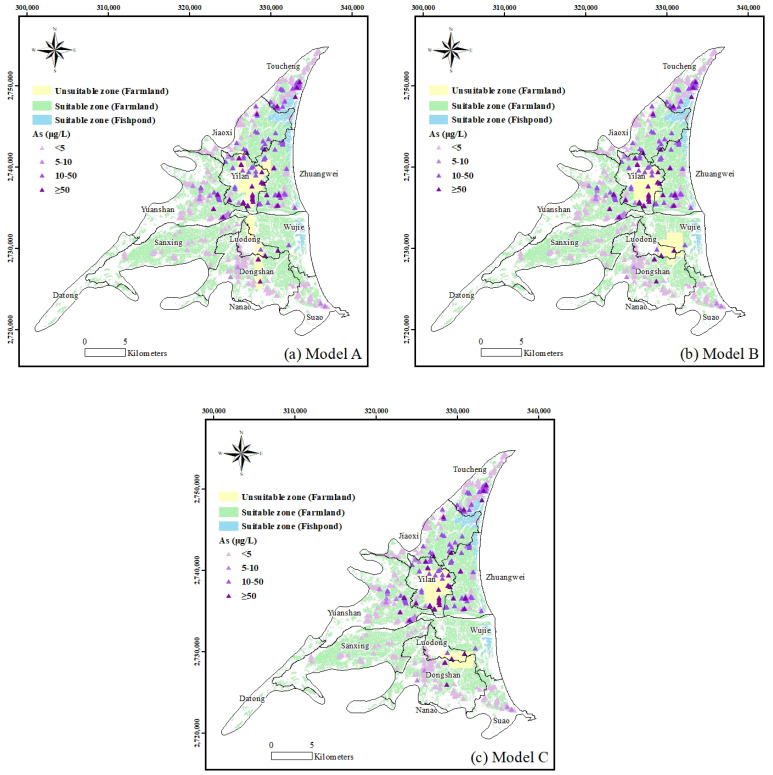
Spatial mapping of the irrigation and aquaculture zones.

Agriculture and aquaculture are the most common types of land usage in the Lan-yang Plain and they are heavily dependent upon groundwater to meet their demands. According to the Council of Agriculture, Taiwan, the acceptable limit for As concentration in irrigation and aquaculture is 50 µg/L. Figure 7 shows the zones that are unsuitable for farmland and fishponds, where the estimated groundwater As concentrations exceed the water quality standards safe for irrigation and aquaculture. These are zones where the groundwater As concentrations are defined as unsafe for irrigation or aquaculture but currently being used for farmlands and fishponds. Land-use practices need to be changed in these regions.

## 4. Conclusions

We performed spatial mapping of the As concentration in groundwater and made a comparison between two distinct approaches: backward propagation neural network (BPNN) and ordinary kriging (OK). The findings show that the BPNN has better prediction performance than the OK method. Subsequently, the BPNN was used to develop spatial maps showing the geographical distribution of As contaminations in the groundwater. The As concentrations obtained using the BPNN approach were then used to develop a spatial map for carcinogenic and noncarcinogenic health risk associated with exposure to arsenic through the drinking of groundwater. For zones with unaccepted HQs and TRs, the promising measures include supply of safe tap water and public education to raise community awareness. The spatial mapping shows the regions unsuitable for farmland and fishponds, as defined by the estimated groundwater As concentrations, exceed the water quality standards for irrigation and aquaculture. Groundwater as a water source should be replaced with the supply of treated, safe surface water, or by using groundwater collected from other areas in regions where the groundwater quality is unsafe for irrigation or aquaculture but is currently being used for farmlands and fishponds. Alternatively, improved land management practices offer promising possibilities to ensure the availability and quality of water for farmlands and fishponds

## Figures and Tables

**Table 1:** Average temperature and rainfall in Lanyang Plain.

Month	1	2	3	4	5	6	7	8	9	10	11	12
**Rainfall (mm)**	230	202	137	126	230	233	140	211	516	702	523	339
**Temperature (°C)**	16.6	17.1	19	21.9	24.7	27.3	28.9	28.6	26.8	23.8	21.1	17.9

**Table 2:** Descriptive statistics of the monitored As concentrations in the Lanyang Plain.

Statistics	As Concentrations (μg/L)			
	Total	Dataset A	Dataset B	Dataset C
Well number	921	307	307	307
Average	11.9	11.56	11.22	12.94
Median	0.89	0.45	1	1
Standard deviation	45.26	42.58	35.97	55.11
Relative standard deviation	3.80	3.68	3.21	4.26
Skewness	9.07	7.30	6.00	10.06
Minimum	0.45	0.45	0.45	0.45
Maximum	776.25	489.78	338.84	776.25
Percentiles				
50th	0.89	0.45	1	1
82.75th	10	10.72	10	10

**Table 3:** Fitted parameters for the exponential model.

Training Dataset	*c*_0_	*c*	*a*
BC	0.01	0.05	10,000
AC	0.015	0.05	15,000
AB	0.02	0.05	15,000

**Table 4:** Parameters for building the BPNN.

Input Node Number	2
1st hidden layer neuron number	2, 4, 6, 8…50
2nd hidden layer neuron number	0, 2, 4, 6…50
output layer neuron number	1
activation function of 1st hidden layer neuron	hyperbolic tangent sigmoid
activation function of 2nd hidden layer neuron	hyperbolic tangent sigmoid
activation function of output layer	Pureline
initialization of weighting factors	random value between −1 and 1
initialization of bias	random value between −1 and 1
convergence criteria	mse = 10^−2^

**Table 5:** Average coefficients of determination (R^2^) and root mean square error (RMSE) used in each BPNN model.

BPNN Structure		R^2^				RMSE				BPNN Structure		R^2^				RMSE			
		Training		Validation		Training		Validation				Training		Validation		Training		Validation	
		(Average)		(Average)		(Average)		(Average)				(Average)		(Average)		(Average)		(Average)	
(2,2,1)	A	0.37	0.39	0.45	0.38	0.58	0.57	0.54	0.57	(2,2,2,1)	A	0.21	0.29	0.24	0.28	0.65	0.62	0.64	0.62
	B	0.36		0.36		0.59		0.58			B	0.42		0.40		0.56		0.56	
	C	0.42		0.34		0.56		0.59			C	0.23		0.20		0.64		0.65	
(2,4,1)	A	0.43	0.43	0.50	0.43	0.55	0.55	0.52	0.55	(2,4,4,1)	A	0.48	0.48	0.52	0.44	0.53	0.53	0.51	0.55
	B	0.45		0.45		0.54		0.54			B	0.50		0.45		0.51		0.54	
	C	0.41		0.33		0.56		0.60			C	0.44		0.35		0.55		0.59	
(2,6,1)	A	0.49	0.49	0.54	0.46	0.52	0.52	0.50	0.54	(2,6,6,1)	A	0.60	0.58	0.60	0.53	0.46	0.47	0.47	0.50
	B	0.46		0.45		0.54		0.54			B	0.53		0.49		0.50		0.52	
	C	0.52		0.41		0.50		0.57			C	0.61		0.50		0.46		0.51	
(2,8,1)	A	0.54	0.53	0.56	0.50	0.50	0.50	0.49	0.52	(2,8,8,1)	A	0.56	0.58	0.56	0.52	0.49	0.47	0.49	0.50
	B	0.53		0.51		0.50		0.51			B	0.64		0.58		0.44		0.47	
	C	0.54		0.42		0.50		0.56			C	0.54		0.43		0.49		0.55	
(2,10,1)	A	0.48	0.52	0.53	0.51	0.53	0.50	0.50	0.51	(2,10,10,1)	A	0.63	0.62	0.60	0.55	0.44	0.45	0.46	0.49
	B	0.51		0.50		0.51		0.52			B	0.59		0.54		0.47		0.50	
	C	0.58		0.49		0.47		0.53			C	0.64		0.52		0.44		0.51	
(2,12,1)	A	0.56	0.58	0.55	0.51	0.49	0.48	0.50	0.50	(2,12,12,1)	A	0.64	0.63	0.58	0.54	0.44	0.45	0.64	0.50
	B	0.59		0.50		0.47		0.48			B	0.57		0.51		0.48		0.56	
	C	0.58		0.49		0.48		0.53			C	0.68		0.53		0.42		0.65	

**Table 6:** Averaged coefficients of determination and root mean square errors of the validation datasets obtained with the two methods.

		A	B	C	Average
OK	R^2^	0.54	0.48	0.46	0.49
	RMSE	0.52	0.54	0.56	0.54
BPNN	R^2^	0.60	0.54	0.52	0.55
	RMSE	0.46	0.50	0.51	0.49

**Table 7:** Parameters used in calculating carcinogenic and noncarcinogenic health risk.

Parameters (Units)	Parameter Characteristics
C (µg/L)	-
ED (year)	30 ^a^
EF (day/year)	365 ^a^
IR (L/day)	1.4 ^b^
BW (kg)	64.5 ^b^
AT (day)	79.0×365 = 28,835 ^a^
$\mathrm{RfD}\;(\frac{µg}{\mathrm{kg}\cdot \mathrm{day}})$	0.3 ^c^
$\mathrm{CSF}\;(\frac{\mathrm{kg}\cdot \mathrm{day}}{\mathrm{mg}})$	1.5 ^c^

^a^ Liang et al. [12]. ^b^ Compilation of Exposure Factors (2008). ^c^ USEPA (Retrieved from: https://cfpub.epa.gov/ncea/iris_drafts/atoz.cfm, accessed on 28 October 2021).

## Data Availability

Not applicable.

## References

[1] EPB (1997). Survey of Arsenic Contents of Drinking Water (Surface Water and Groundwater) in YiLan County.

[2] EPB (1998). Survey of Arsenic Contents of Drinking Water (Surface Water and Groundwater) in YiLan County.

[3] EPB (1999). Survey of Arsenic Contents of Drinking Water (Surface Water and Groundwater) in YiLan County.

[4] World Health Organization (WHO) (1993). Guidelines for Drinking Water Quality: Recommendations.

[5] International Agency for Research on Cancer (IARC) (2012). A Review of Human Carcinogens: Arsenic, Metals, Fibers, and Dusts.

[6] Tseng W.P. (1977). Effects and dose-response relationships of skin cancer and blackfoot disease with arsenic. Environ. Health Perspect..

[7] Chen C.J., Chuang Y.C., Lin T.M., Wu H.Y. (1985). Malignant neoplasms among residents of a blackfoot disease-endemic area in Taiwan: High-arsenic artesian well water and cancers. Cancer Res..

[8] Hsueh Y.M., Wu W.L., Huang Y.L., Chiou H.Y., Tseng C.H., Chen C.J. (1998). Low serum carotene level and increased risk of ischemic heart disease related to long-term arsenic exposure. Atherosclerosis.

[9] Tseng C.H., Tai T.Y., Chong C.K., Tseng C.P., Lai M.S., Lin B.J., Chiou H.Y., Hsueh Y.M., Hsu K.H., Chen C.J. (2000). Long-term arsenic exposure and incidence of non-insulin-dependent diabetes mellitus: A cohort study in arseniasis-hyperendemic villages in Taiwan. Environ. Health Perspect..

[10] Liang C.P., Wang S.W., Kao Y.H., Chen J.S. (2016). Health risk assessment of groundwater arsenic pollution in southern Taiwan. Environ. Geochem. Health.

[11] Lee J.J., Jang C.S., Wang S.W., Liu C.W. (2007). Evaluation of potential health risk of arsenic-affected groundwater using indicator kriging and dose response model. Sci. Total Environ..

[12] Liang C.P., Chien Y.C., Jang C.S., Chen C.F., Chen J.S. (2017). Spatial analysis of human health risk due to arsenic exposure through drinking groundwater in Taiwan’s Pingtung Plain. Int. J. Environ. Res. Public Health.

[13] Liang C.P., Chen J.S., Chien Y.C., Chen C.F. (2018). Spatial analysis of the risk to human health from exposure to arsenic contaminated groundwater: A kriging approach. Sci. Total Environ..

[14] Jang C.S., Chen C.F., Liang C.P., Chen J.S. (2016). Combining groundwater quality analysis and a numerical flow simulation for spatially establishing utilization strategies for groundwater and surface water in the Pingtung Plain. J. Hydrol..

[15] Liang C.P., Hsu W.S., Chien Y.C., Wang S.W., Chen J.S. (2019). The combined use of groundwater quality, drawdown index and land use to establish a multi-purpose groundwater utilization plan. Water Resour. Manag..

[16] Chowdhury M., Alouani A., Hossain F. (2010). Comparison of ordinary kriging and artificial neural network for spatial mapping of arsenic contamination of groundwater. Stoch. Environ. Res. Risk Assess..

[17] Purkait B., Kadam S., Das S. (2008). Application of Artificial Neural Network Model to Study Arsenic Contamination in Groundwater of Malda District, Eastern India. J. Environ. Inform..

[18] Cho K.H., Sthiannopkao S., Pachepsky Y.A., Kim K.W., Kim J.H. (2011). Prediction of contamination potential of groundwater arsenic in Cambodia, Laos, and Thailand using artificial neural network. Water Res..

[19] Jeihouni M., Delirhasannia R., Alavipanah S.K., Shahabi M., Samadianfard S. (2015). Spatial analysis of groundwater electrical conductivity using ordinary kriging and artificial intelligence methods (Case study: Tabriz plain, Iran). Geofizika.

[20] Jia Z., Zhou S., Su Q., Yi H., Wang J. (2017). Comparison study on the estimation of the spatial distribution of regional soil metal (loid)s pollution based on kriging interpolation and BP neural network. Int. J. Environ. Res. Public Health.

[21] Jean J.S., Bundschuh J., Chen C.J., Lin T.F., Chen Y.H. (2010). The Taiwan Crisis: A Showcase of the Global Arsenic Problem.

[22] Liu C.W., Wu M.Z. (2019). Geochemical, mineralogical and statistical characteristics of arsenic in groundwater of the Lanyang Plain, Taiwan. J. Hydrol..

[23] Nunes Silva I., Hernane Spatti D., Andrade Flauzino R., Liboni L.H.B., dos Reis Alves S.F. (2017). Artificial Neural Networks a Practical Course.

[24] Journel A.G., Huijbregts C.J. (1978). Mining Geostatistics.

[25] United States Environmental Protection Agency (USEPA) (2005). Guidelines for Carcinogen Risk Assessment.

[26] United States Environmental Protection Agency (USEPA) Integrated Risk Information System (IRIS) Assessment. https://cfpub.epa.gov/ncea/iris_drafts/atoz.cfm.

